# Two-Stage Genome-Wide Search for Epistasis with Implementation to Recombinant Inbred Lines (RIL) Populations

**DOI:** 10.1371/journal.pone.0115680

**Published:** 2014-12-23

**Authors:** Pavel Goldstein, Abraham B. Korol, Anat Reiner-Benaim

**Affiliations:** 1 Department of Statistics, University of Haifa, Haifa, 3498838, Israel; 2 Department of Evolutionary and Environmental Biology and Institute of Evolution, University of Haifa, Haifa, 3498838, Israel; MOE Key Laboratory of Environment and Health, School of Public Health, Tongji Medical College, Huazhong University of Science and Technology, China

## Abstract

**Objective and Methods:**

This paper proposes an inegrative two-stage genome-wide search for pairwise epistasis on expression quantitative trait loci (eQTL). The traits are clustered into multi-trait complexes that account for correlations between them that may result from common epistasis effects. The search is done by first screening for epistatic regions and then using dense markers within the identified regions, resulting in substantial reduction in the number of tests for epistasis. The FDR is controlled using a hierarchical procedure that accounts for the search structure. Each combination of trait and marker-pair is tested using a model that accounts for both statistical and functional interpretations of epistasis and considers orthogonal effects, such that their contributions to heritability can be estimated individually. We examine the impact of using multi-trait complexes rather than single traits, and of using a hierarchical search for epistasis rather than skipping the initial screen for epistatic regions. We apply the proposed algorithm on *Arabidopsis* transcription data.

**Principal Findings:**

Both epistasis detection power and heritability contributed by epistasis increased when using multi-trait complexes rather than single traits. Epistatic effects common to the eQTLs included in the complexes have higher chance of being identified by analysis of multi-trait complexes, particularly when epistatic effects on individual traits are small. Compared to direct testing for all potential epistatic effects, the hierarchical search was substantially more powerful in detecting epistasis, while controlling the FDR at the desired level. Association in functional roles within genomic regions was observed, supporting an initial screen for epistatic QTLs.

## Introduction

Epistasis, or interaction between genes, is a fundamental phenomenon that is believed to play an important role in diverse fields of biology, including quantitative genetics and genomics of complex diseases, gene regulatory networks and biochemical pathways and sex evolution, as well as genome and proteome evolution. Despite the growing interest in searching for epistatic interactions, there is no consensus as to the best strategy for their detection within a genome-wide scan [1]. The problem is particularly complicated in the case of expression QTL (eQTL) analysis, where the expression of a particular gene is considered as a single trait. Microarray data is used to monitor expression levels and epistasis is searched for among numerous combinations of expression traits and genetic markers on the chromosomes. The number of tests involved, which equals the number of genes (traits) for which expression is profiled multiplied by the number of eQTL pairs, is enormous even when considering only pairwise eQTL interactions. A dimension reduction stage can be of help, nevertheless the type I error across all tests must be controlled. A popular strategy for reducing the number of tests for epistasis is to first screen for one-locus effects, in which the genotype of a particular locus has a marginal effect on a particular expression trait. Next, for each selected combination of trait and marker pair, epistasis is searched for that trait only within the identified chromosomal regions [2]. This strategy implies that an epistatic interaction is tested for only if there are main effects. However, it has been shown that epistasis may appear also in cases for which marginal loci effects are absent [2], [3] suggesting that screening by main effects may lead to overlooking of some of the interactions. The authors tested three-way epistasis and discovered three-locus interactions that were not apparent from the two-way epistasis. [2] point out that the situation in which epistasis controls variation in quantitative traits has been relatively neglected in research, even though it possesses a significant biological meaning and may contribute substantially to the understanding of complex gene networks.

A more recent approach takes into account the strong correlations between neighboring markers in the new generation of ultra-dense genetic maps, enabling a dramatic reduction in the number of tests to be performed. Such an approach is also adopted by [4], suggesting that SNPs that lie in cis to a particular gene are potential SNPs for epistatic effects. Thus an epistasis searching algorithm can start with matching pairs of SNP’s based on prior knowledge from biological experiments regarding the associations between the SNP’s. Additionally, skipping spaces with a length of 10 cM during two-dimensional epistasis scan, and using representative regional markers rather than considering all possible pairs of markers, may improve the accuracy of the search [5].

A widely used approach for reducing the dimension of gene expression data is identifying groups of genes that share similar expression profiles. For instance, instead of analyzing each individual gene expression trait for the purpose of eQTL mapping, treating principal components of the individual gene expression as traits has been shown to improve the performance of eQTL analysis [6]. Considering correlated traits as multi-trait complexes has been shown to increase QTL detection power, mapping resolution and estimation accuracy [7]. It was also found that for a given set of quantitative traits, as the number of considered traits increases, the QTL heritability (the proportion of variance explained by genetic effects) increases as well. These findings make biological sense, as a single mutation typically affects multiple related traits [8]. Thus traits within a particular group may be subjected together to pleiotropic effect of the same epistatically interacting loci as well as other genetic factors contributing to trait variation. Taking advantage of the correlation between phenotypes for the purpose of increasing power has been given attention also for other types of genomic studies, such as GWAS. For instance, the correlation can be accounted for when estimating genotypic effects by several types of SNP-phenotype association models such as mixed models, GLM or GEE [9]. Furthermore, when multiple phenotypes of various scales measure the same underlying traits, [10] offer a scaled model for estimating the effects of SNPs in case-control studies. Zhang et al [11] refer to epistasis as co-expressed genes that map to a common set of markers, and use a Bayesian partition approach for detecting such effects.

We propose an inegrative two-stage approach for searching for pairwise epistasis, which pursues the potential power benefits of both initial screening and the use multi-trait complexes. In the first stage, clusters of traits are constructed, and the first principal component of each cluster is regarded as a single (complex) trait in eQTL analysis. Here the clusters are formed using the Weighted Gene Co-Expression Network Analysis (WGCNA) algorithm, a clustering-based framework for building gene expression networks [12], [13]. They offer a new dissimilarity measure, based on a topological overlap matrix (TOM) for converting genes’ co-expression into a degree of connectedness, and then implement hierarchical clustering that includes a dynamic top-down branch cutting method for detecting gene modules, depending on their shape. The second stage performs a hierarchical search for epistasis. It starts with an initial “rough” scan for pairwise interactions among “framework” markers, which are markers positioned at relatively distant loci of each other. Next, a higher resolution scan only within the identified regions is performed. In both steps of the scan, each combination of marker pair and trait complex is tested for epistasis, interpreted as the interaction in a two-way analysis of variance model.

The proposed framework allows flexibility in choosing the modeling approach of epistasis. In this paper we use the Natural and Orthogonal Interactions (NOIA) model [14] to test the null hypothesis that there is no epistasis, for each combination of marker pair and trait. We find this model approach appealing for several reasons. First, the NOIA framework generates orthogonal estimates for main effects and epistasis. Orthogonality is one of the desirable properties when modeling genetic effects, particularly when the main focus is in finding “pure” epistatic effects that are distinctly differentiated from other effects. Second, the epistasis effect in the NOIA model can be easily and straightforwardly interpreted as a statistical interaction term in an ANOVA model. Alternative models offer different, more complex, interpretations of epistasis. The multiple locus linkage analysis proposed by [15] assesses the joint significance of multiple loci in affecting a quantitative trait. For each combination of trait and a set of pre-selected loci, Bayesian probabilities are combined to form the probability that all loci are linked to the expression trait. The model of genetic interaction networks proposed by [16] produces a network that exhibits between-locus interactions as well as loci–to-phenotype effects. Third, the construction of the NOIA model allows flexible definitions in accordance with the population in hand, as detailed in the Methods section. In this paper we work out a modified form of the model that matches our RIL population data, in which the dominance effect is not present. The model can also be maneuvered to suit other populations, such as F2, for which the NOIA model is originally defined in [14]. Fourth, [14] refers to the potential confounding effect between allele frequencies and genotypic effects. A simulation study performed in [14] indicated that the NOIA statistical model enables describing multi-locus genetic interactions regardless of the allele frequencies at the trait loci, thus avoiding estimation biases due to segregation distortion and sampling errors. In addition, it is argued there that the NOIA model generates orthogonal effects, regardless of the genotypic frequencies in the population.

An important issue when testing a large amount of hypotheses is the control of type I error across all tests. We suggest controlling the False Discovery Rate (FDR), i.e. the expected proportion of false epistatic effects discoveries, among all discovered epistatic effects. The initial screen for epistasis followed by a higher resolution scan within the selected regions implies that the tests performed within the two steps are organized in a hierarchical manner. The hierarchical FDR controlling procedure offered by [17], which accounts for the hierarchical organization of tests, is used in this work within the proposed algorithm. This direction for addressing the large multiplicity problem is also suggested in [18], explaining that if lower or higher levels of resolution are of interest, then it is possible to use hierarchical multiple testing methods that test more than one level of resolution simultaneously. The hierarchical procedure offered by [17] was implemented in [19] within a gene expression study that involved a similar screening stage for the purpose of finding genes of which expression correlated with behavioral phenotypes. When the findings of interest were particularly scarce, as is typically the case when epistatic effects are of concern, the procedure was shown to have an advantage in power over an alternative method, termed “subset selection”, which did not account for the hierarchical organization of the hypotheses. The control the FDR by the hierarchical procedure is theoretically established by [17] when the two scanning steps are independent. Simulation studies have shown FDR control for the hierarchical testing also under weak dependence [19].

We evaluate the performance of the proposed two-stage algorithm by examining the contribution of using multi-trait complexes rather than single traits, and of using a hierarchical search for epistasis, rather than skipping the initial screen for epistatic regions. We first do so by implementing the proposed hierarchical testing on single traits and on trait complexes, and implementing the modeling of epistasis with and without an initial screen for potential epistatic regions. We compare these alternatives by several performance measures including the contribution of the epistatic effect to the traits’ heritability, the detection power of the procedure and the level of FDR control. The data is simulated under several configurations of number of individual QTL effects and size of epistatic effect. In addition, we compare the proposed algorithm to an alternative QTL detection method that is based on the popular Bayesian interval mapping approach and applies a Markov chain Monte Carlo (MCMC) algorithm for evaluating the posterior of genetic architecture [20], [21]. Since this method does not deal with FDR control, we also assess its power after an FDR adjustment of the resulting posterior probabilities. Furthermore, we employed this method on single traits and on multi-trait complexes. Finally, the proposed algorithm is applied to Arabidopsis thaliana eQTL mapping data, and the results are compared to those obtained by analysis of single traits. The data on a Recombinant Inbred Line (RIL) population is used due to the simplicity of homozygous lines, yet the methodology can be implemented to other types of mapping populations using an appropriate formulation of the NOIA model.

## Materials and Methods

### The NOIA model defined for two-locus epistasis in RIL population

Consider a locus A and a quantitative trait affected by this locus. [22] proposed to link *G*, the vector of expected phenotypic values of the two possible alleles, to E, the vector of genetic effects, by *G = S E*, where S is a genetic-effect design matrix. Due to the absence of heterozygotes in RIL populations, the dominance effect is eliminated from the model of [14], and then the phenotypes can be expressed by
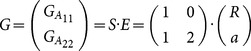
(1)


such that R is a reference point that is interpreted as the mean over the phenotypic values *G_A11_* and *G_A22_*, and a is the additive effect of locus *A*.

Extending the model for two loci *A* and *B*, with genetic-effect design matrices *S_A_* and *S_B_*, respectively, the two-locus vector of genetic effects, *E_AB_*, can be expressed by the matrix product
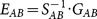
(2)where



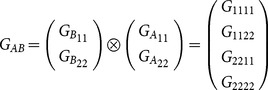
(3)where 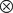 marks the Kronecker product (see also [22] for further demonstration of its use for the NOIA model). The vector 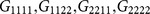 denotes the phenotypic values of the four possible genotypic combinations of the alleles 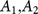 and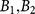, respectively, and
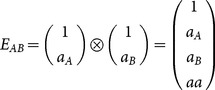
(4)




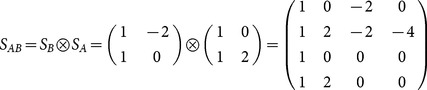
(5)


Where 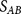 is the genetic-effect design matrix that ensures orthogonality of the model effects and 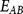 = (1 = intercept, 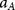 = marginal effect on 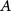, 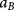 = marginal effect on 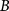, 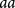  = epistasis) is the vector of genetic effects of the two loci. In this case, NOIA functional formulation, which is concerned here with the effect of two allele substitutions on the trait value, is also an orthogonal statistical formulation, which interprets epistasis as an interaction effect between the loci and provides independent estimates for the effects.

A two-locus statistical model of the effects on for RIL populations may be defined as follows. A genome-wide statistical formulation for the link in (2) can be defined as follows Let 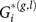
denote the observed gene expression level for trait 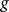 loci-pair 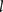 corresponding to markers 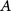 and 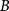 and replicate 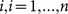. Then for some 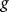 and 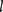, the statistical model can be written algebraically as follows:
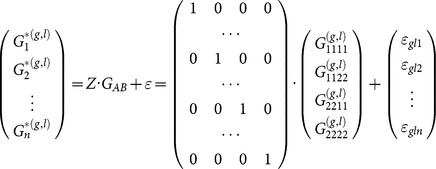





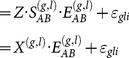
(6)where the 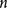 rows of the 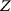 matrix indicate the observed genotypes for the *n* observed expression values and 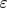 is the vector of errors. The model can be modified for any population by suitable adjustments of 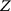, 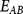 and 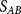.

### The WGCNA clustering algorithm

[12] and [13] suggest a hierarchical clustering framework for building gene expression networks. They introduce a new dissimilarity measure for converting genes’ co-expression into a degree of connectedness - the topological overlap matrix (TOM), which reflects relative inter-connectedness of every two genes and has been found useful in biological networks. [13] present a novel dynamic top-down branch cutting method for detecting gene modules depending on their shape. Compared to the fixed height cutoff method, this approach is shown to be (i) flexible; (ii) capable of identifying nested clusters; (iii) better detects outliers; and (iv) identifies biologically meaningful gene modules. The top-down algorithm starts with a rough segmentation into a small number of large clusters by a static tree cut. Then the accumulation of heights in each cluster is analyzed for identification of typical pattern of fluctuations indicating a sub-cluster structure. Clusters exhibiting this pattern are split. Very small clusters are aggregated to their nearest major clusters to avoid over-splitting. The genes in each final cluster are used to form a unified representation of the cluster that may be termed a meta-gene, or eigen-gene, by taking the first principal component of the genes assigned to the cluster.

Unlike the hierarchical approach, flat partitioning techniques such as k-means and its variants produce independent clusters of genes. We prefer to avoid assuming such independence due to the potential networking relations between genes. In additions, these techniques typically require pre-specifications of the number of clusters, which is an impractical challenge when searching for traits connected by common epistatic effects.

### Trees of hypotheses and hierarchical FDR control

If the hypotheses can be arranged in a form of a tree, a hierarchical procedure to control the FDR across the tree of hypotheses is suggested [17]. In our case, all hypotheses could be arranged in a two-level structure. On the first level are the hypotheses for all combinations of multi-trait complexes and pairs of sparse framework markers. On the second level are the hypotheses for all combinations selected in the first level, this time using “secondary”, more densely positioned markers found near the corresponding framework markers. Generally, under the hierarchical approach, the set of tested hypotheses, 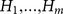, is arranged on a tree with 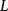 levels. The hypotheses on the first level of the tree have no parental hypotheses; each hypothesis, 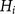 on level 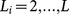 is associated with a single parental hypothesis, indexed by 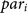, on level 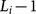. Let 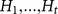 denote the parental hypotheses; then the 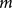 hypotheses can be divided into 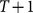 “families”, 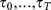, where 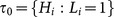 and 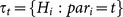, 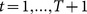. Thus, a “family” is a group of hypotheses having a common “parent” hypothesis. Hypotheses in the same family are tested simultaneously using the FDR controlling linear step-up procedure (hereafter BH) [23], and testing begins with 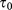. A family of hypotheses on a higher level of the tree is tested only if its parental hypothesis is rejected.

Several types of hierarchical FDR criteria can be defined. A general FDR bound is given by [17]

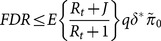
(7)where 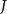 is the number of families tested, 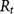 is the total number of discoveries, 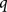 is the level used in the BH procedure, 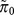 is a weighted mean of the proportion of true null hypotheses in the 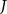 families of hypotheses and 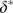 is family-specific multiplicative factor, of which upper bound is shown in to be 1.44 [17].

Since the interest in this work is in all identified epistatic effects, whether by framework or by secondary markers, 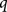 is chosen such that the full-tree FDR is controlled at the desired level using the specific FDR upper bound.
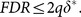
(8)


### A two-stage algorithm for epistasis detection

This work presents an algorithm based on a two-stage procedure for detecting epistatic interactions between pairs of markers across the genome. In the first step, multi-trait complexes are identified by the WGCNA clustering algorithm (using the ‘WGCNA’ R package [24]). The expression traits in each final cluster are used to form a unified representation of the cluster, which may be termed a “meta-trait”, by taking the first principal component of the expression traits from every cluster.

Dependence between neighboring DNA regions, arising from tight linkage of the markers, motivates a hierarchical search which starts with an initial screen for epistatic regions followed by a more focused search. Using sparse framework markers distant about 10 cM from each other, a rough scan is performed for every multi-trait complex (or meta-trait) obtained in the preceding dimension-reduction step. In the second step, only the combinations of meta-traits and framework marker pairs showing epistasis (regardless of the main effects) are further explored. Now the scan is performed with higher resolution, using “secondary”, more densely positioned markers found near the corresponding framework markers. The NOIA model is employed individually on each tested combination in each step, in order to test for epistasis. Due to the orthogonality property, each identified effect is independent of the rest of the effects in the model, such that the heritability of an effect, namely the contribution of the effect to the total gene expression variance, can be obtained.

**Figure 1:**
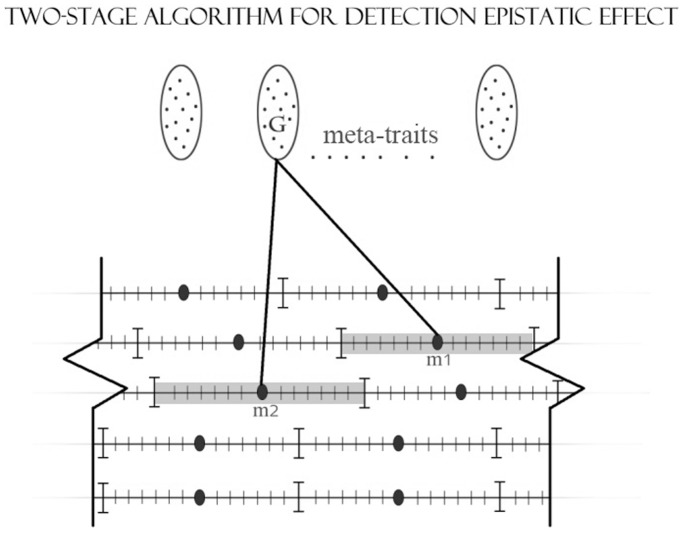
A schematic description of the two-stage epistasis detection algorithm. Meta-traits with the corresponding traits are presented as ellipses with dots. Below them a partial genetic map of five *Arabidopsis* chromosomes is zoomed in. Filled circles and short vertical lines denote “framework” and “secondary” markers, respectively, and long vertical lines denote borders of sparse marker regions. The figure describes discovery of epistasis for the combination of meta-trait *g*, sparse marker pair 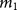 and 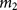 in the first step, and their “secondary” markers (in grey) in the second step.

The full algorithm is formally described as follows. Fig. 1 provides a visual representation of the two stages.

1. Identification of multi-trait complexes - using WGCNA clustering.
2. Identification of epistasis - using the two-step hierarchical testing procedure:
  - Let 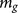 be the number of meta-traits obtained in step 1 and let 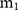 be the number of pairs of framework markers. Then for each combination of meta-trait 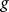, 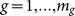 and pair of framework markers 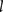, 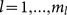, the NOIA model as formulated in (6) is used to test the null hypothesis 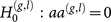, where 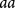 is the epistatic effect defined in (6).
  - For each rejected hypothesis, the hypotheses within its family are tested for the combinations of the corresponding meta-trait with all corresponding pairs of secondary markers.

### Alternative approach for epistatic eQTL mapping: Bayesian model selection

Consider a single trait with phenotypic trait values 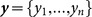 and marker genotypes 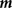. The Bayesian model selection framework proposed in [21] partitions the entire genomic map into 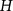 loci denoted by the vector 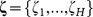 and assumes that the possible QTLs occur at these fixed positions. 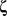 includes not only the marker positions but also points between markers. Thus, the genotypes 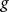 of an unobserved marker included in loci 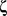 are unobservable, but their probability distribution, denoted by 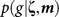, can be estimated using the multipoint method [25].

Let 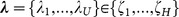 be the positions of 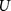
putative QTLs. Then a relationship between 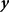 and 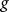 can be expressed by
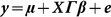
(9)where 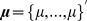 is the overall trait mean, 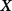 is a design matrix, 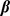 denotes the vector of all main genetic effects and pairwise interactions for the 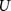 potential QTLs and 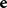 is the vector of independent normal errors, each with mean 0 and variance 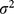. Let 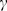 be a 0–1 variable defined for each effect, indicating if the corresponding effect is included in the model. Then the genetic architecture is specified by 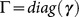, where 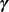 is a matrix of which diagonal contains the 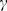 indicators, implying also the number of QTLs in the model. The likelihood of the model may be denoted by 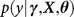, with parameter vector 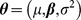. Then the posterior distribution can be expressed by



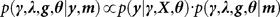
(10)The parameters of interest, 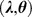, are estimated by profiling the likelihood function and accounting for the genetic architecture described by 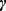. The posterior Bayesian parameter estimates are developed and obtained in [21] by using MCMC simulations. At each iteration, the full Gibbs sampler generates the indicator variables matrix γ from its conditional posterior distribution. The posterior inclusion probability 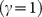 for each locus is estimated by calculating its frequency in the posterior samples. Each locus may be included in the model through its main effects and/or epistasis with another locus. The larger the effect size for a locus, the more frequently the locus occurs in the samples. Taking the prior probability into consideration, Bayes factors (BF) can be used to show evidence for inclusion against exclusion of a locus. The Bayes factor for comparing two genetic structures 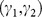 is defined by
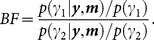
(11)


The procedure in [21] uses *BF* = 3 based on the findings of [26] as a threshold of significance. For two QTLs 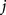 and 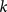 with indicators for the individual effects 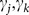 and epistasis indicator 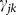, the inclusion prior probability for individual effects is defined as 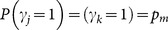 and for epistasis inclusion:
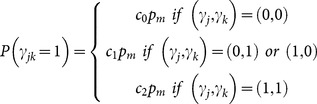
(12)where here 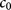, 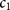 and 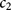 correspond to prior weights.

### Simulation study

A simulation study was conducted in order to assess (i) an improvement in epistasis detection power and in heritability assigned to epistasis as a result of using multi-trait complexes and of using hierarchical testing, and (ii) the FDR control level achieved by the procedure under the dependence structure typical to the tree of hypotheses tested here. Intensities were generated from multivariate normal distribution with mean similar to the observed intensity mean and with standard deviation of 2.5, which allowed variability in power. Non-epistatic clusters were generated with a correlation of 0.4 between the traits. Genotypic information was based on real markers on the *Arabidopsis* map (see Section 2.5) at twelve regions around framework markers in three chromosomes. Epistatic effects were “implanted” on the actual markers at absolute levels ranging from 0.19 to 0.21, in accordance with effects observed for single traits included in trait-complexes that were found to have epistatic effects. These levels also allowed flexibility in power between single-trait analysis and meta-trait analysis. All possible main effect and epistasis combinations were examined in the simulation (epistasis with one main effect, epistasis with two main effects, epistasis only). Gene expression intensities were simulated according to a four-cluster structure, each cluster containing fifteen traits. Two of the four clusters contained traits with epistasis attributed to pairs of markers emerging from the partial *Arabidopsis* map. For a cluster simulated with epistasis, 13 (86%) of its traits had either inter-chromosomal or intra-chromosomal epistatic effects. The two remaining non-epistatic traits represented error generated by non-specific cluster identification. All effects were signified by NOIA model coefficients, where the interaction coefficient reflected the size of the epistatic effect. For the epistatic traits, the number of main eQTL effects was one, two or none. The coefficients of all effects were set to levels that allowed flexibility in the contribution to heritability and the detection power.

The proposed algorithm was implemented for all configurations of simulated data, starting by calculating the first principal component of the gene-expression traits included in each cluster. Next, for the first step of the hierarchical testing procedure, a scan for epistasis was performed by fitting a NOIA model (by the ‘noia’ R Package [27]) to each combination of meta-trait and twelve sparse framework markers in the original *Arabidopsis* RIL map. In the second step, the scan was performed with higher resolution on six “secondary”, more densely positioned markers neighboring each of the framework markers identified in the first step. The proposed approach was compared to the Bayesian model selection approach of [21]. The method is implemented using the qtlbim R package [28]. We used the prior inclusion probability for epistasis defined in (12) in accordance with the simulated number of main effects in addition to epistasis. Probability of 0.5 was used for the actual simulated scenario, and probability of 0.25 was given for the two other possible scenarios.

The power of the test for epistasis was estimated by the average across all simulations of the proportion of detected epistatic traits/meta-traits relative to the total number of epistatic traits/meta-traits. The contribution of epistasis to heritability was estimated by the average across all simulations of the proportion of variance explained by epistasis. FDR was estimated by the average across all simulations of the proportion of falsely identified traits/meta-traits among all identified traits/meta-traits. 200 simulations were used to obtain small enough standard errors. For comparison, (i) the hierarchical testing procedure was also implemented on single traits, and (ii) the analysis on meta-traits was also implemented directly, skipping the initial screen, and the BH procedure was used in order to control the FDR.

### The Arabidopsis data

Gene expression data was downloaded from the TAIR database website (ftp://ftp.arabidopsis.org/home/tair/Microarrays/analyzed_data/affy_data_1436_10132005.zip). This data contains intensities for 22,810 traits from all five chromosomes of the *Arabidopsis thaliana* genome. A sample of 211 RIL population individuals derived from a cross between two inbred accessions, Bayreuth-0 (Bay-0) and Shahdara (Sha), was used. Transcript (mRNA) levels were quantified using Affymetrix whole-genome microarrays with two replications (arrays) for each individual [29]. The corresponding genetic map, consisting of 579 molecular markers, was obtained from http://elp.ucdavis.edu/data/analysis/211_RILs_SFP_map/211_RILs_SFP_map.html. The genotypic data was preprocessed using the MultiPoint software (http://www.multiqtl.com) for the purpose of eliminating non informative, overlapping markers and those markers that cause local neighborhood instability in the map[30]. In total, 493 markers remained for the analysis. The gene expression data was preprocessed by the Variance Stabilization Normalization method [31]. Traits with essentially no expression (15,566 traits) were filtered out by the EM algorithm for a mixture of univariate normal distributions (using the ‘mixtools’ R package), leaving 7244 traits for the analysis.

## Results

### 

#### Simulation study

The proposed algorithm was implemented on simulated data containing epistatic effects. The results are given here for negative epistatic effects and positive main effects, as also used in other cases [32] within NOIA modeling, but similar results were obtained for all other sign combinations (not shown). Main effect coefficients were set to 0.4. The FDR controlling procedure was used at level 0.2 in order to enable some power for the single-trait analysis.

**Figure 2:**
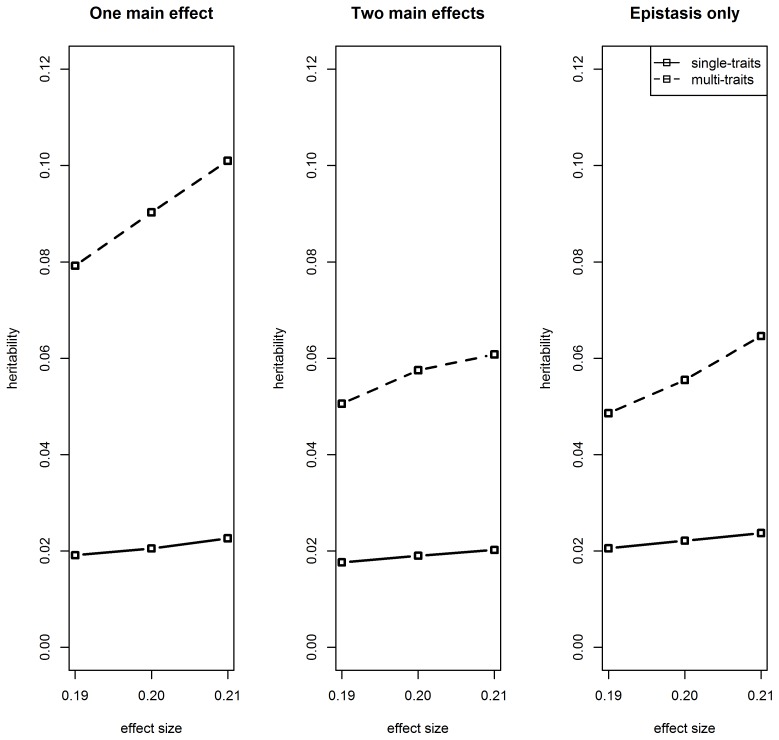
Contribution of epistasis to trait heritability – simulated results. Heritability, the proportion of variance explained by the epistatic effect, is plotted against the epistatic effect size. The solid lines mark the heritability for single trait analysis, and dashed lines mark the heritability for meta-trait analysis. Standard error was <0.003 for single traits and <0.025 for meta-traits.

**Figure 3:**
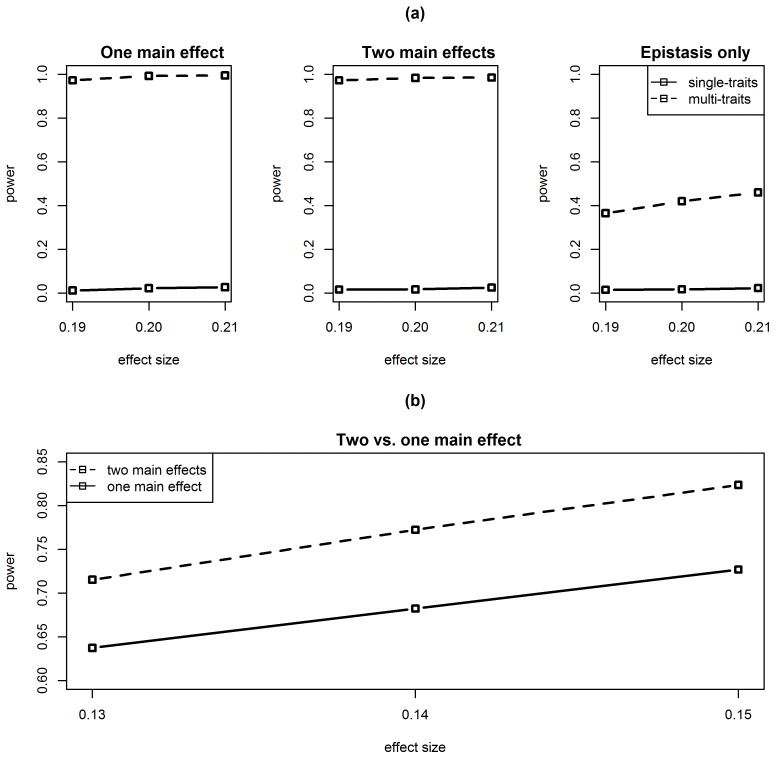
Epistasis detection power – simulation results. Power, estimated by the proportion of epistatic effects detected by the procedure, is plotted against the epistatic effect size. Standard error was <0.003 for single traits and <0.025 for meta-traits. (a) Power levels are saturated for the multi-trait complexes analysis on models with main effects, and thus lower epistatic effects are used in Fig. 3b. (b) Reduced sizes of epistatic effects for the multi-trait complexes analysis, which generate non-saturated power levels, are examined on models with main effects. b. Epistasis detection power for the meta-traits analysis on models with main effects - simulation results. Power, estimated by the proportion of epistatic effects detected by the procedure, is plotted against the epistatic effect size. Standard error was <0.003 for single traits and <0.025 for meta-traits.

As can be seen by the estimated epistatic contribution to heritability (Fig. 2), epistatic effects on meta-traits explained substantially more phenotypic variation of gene expression within the mapping population than effects on single traits. A particularly high contribution was obtained when one of the two eQTLs in the interaction was also a main effect. Furthermore, the search for epistasis within single traits achieved a very low power, while the search within meta-traits achieved far more power, particularly when there was at least one main effect in the model (Fig. 3a). Accordingly, the FDR was controlled at the very low level of 0.0002 when single traits were analyzed and at a higher level of 0.04 when meta-traits were analyzed. Due to the power saturation obtained by the meta-traits analysis for the models containing main effects, the difference in power between the one main effect case and the two main effect case is not visible in Fig. 3a (see the two leftmost plots). Thus lower epistatic effects were used in order to specifically assess the power for these two cases for meta-traits analysis. As can be seen in Fig. 3b, the procedure is more powerful in the case of two main effects compared to the case of one main effect. This may result from the decreased residual variance at the presence of two main effects. It can also explain why most epistatic findings from analyzing the Arabidopsis data (see next sub-section) contain two main, and also supports the rule of thumb for finding epistasis by first screening for traits affected by single markers. Interestingly, while heritability for the meta-trait analysis does not much vary between the main effect configurations, the power of the procedure, and thus the significance of the finding, varies considerably in accordance with the proportion of residual variance.

Next, the impact of the hierarchical search for epistasis on meta-traits was examined. Results are shown in Table 1 for epistatic effect size 0.12, which allowed flexibility in power between hierarchical search and direct search. The advantage in power of the two-step procedure over direct testing of all combinations is clearly evident for either one or two main effects in the model. Power was very low for the epistatic effect used here when there were no main effects (not shown). The FDR was controlled at somewhat lower levels when the hierarchical search was used, in consistence with previous findings [19]. The contribution of epistasis to heritability does not seem to be affected by the type of search. Yet the contribution is mildly higher for the case of one main effect compared to the case of two main effects, consistently with the finding in Fig. 2.

**Table 1:** Direct testing vs. hierarchical testing for epistasis.

Testingapproach	Num. of Eqtlmain effects	Power	Heritability			FDR
			Total	Due to maineffects	Due toepistasis	
Direct	one	.4468			.0272	.0182
	two	. 3776			.0204	.0120
Hierarchical	one	.5812	0.4987	0.4684	.0.0303	.0175
	two	.5152	0.615	0.5945	.0205	.0110

Meta-trait analysis – simulation results. Distribution across effects of heritability is also shown for the hierarchical approach. Standard error is <0.02 for power, <0.0007 for heritability and <0.0009 for FDR.

Examining the distribution of the total heritability across the main effects and epistasis, it can be seen in Table 1 that in the case of epistasis with only one main effect in the model, the contribution to heritability by the effects is enhanced, compared to the case of epistasis with two main effects. It may be a result of the principal component rotation, which seems to better identify the effects when there are fewer of them. Thus in the case of one main effect, the benefit in contribution to heritability is higher for both the main effect (about 47%) and the epistatic effect (about 3%), compared to the benefit for each main effect (about 30% in average) and epistasis (about 2%) in the cases of two main effects. Yet, the principal component transformation is challenged in identifying an epistatic effect that is not accompanied by main effects and is very small relative to the residual variance.

**Figure 4:**
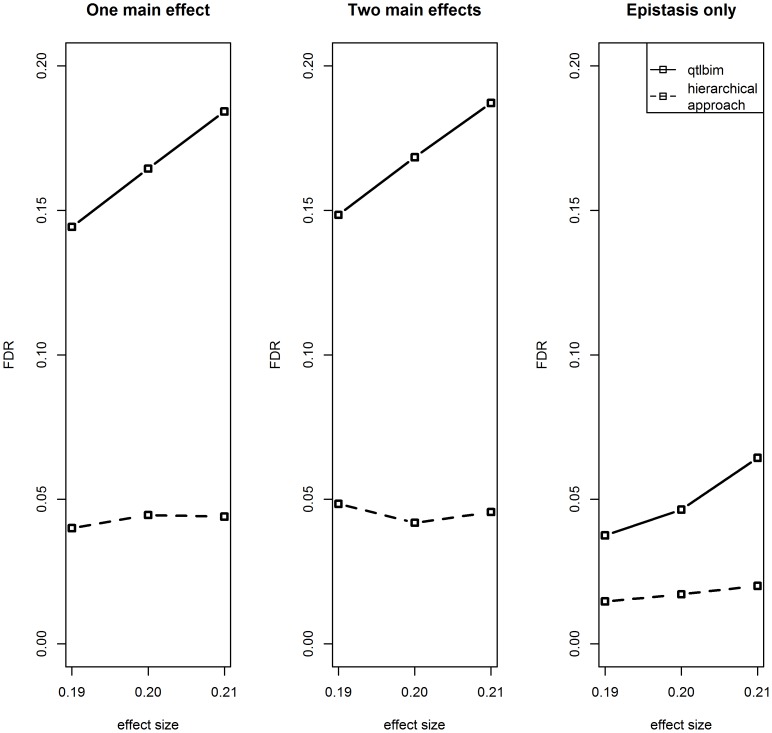
FDR comparison between QTLBIM and the hierarchical testing approach for meta-traits analysis – simulation results. FDR, estimated by the average proportion of erroneously identified epistatic effects among all identified epistatic effects, is plotted against the epistatic effect size. The solid lines mark the FDR obtained by the QTLBIM method, and the dashed lines mark the FDR obtained by the proposed hierarchical approach. Standard error was <0.025 for single traits and <0.003 for meta-traits.

Next, the performance of the Bayesian selection method was compared to our approach (Table 2). Similarly to our method, the search for epistasis within single traits achieved very low power. When searching for epistasis on multi-trait complexes constructed by WGCNA clustering, both methods achieved similar power. However, as can be seen in Fig. 4, while the proposed method controlled the FDR at a level lower than the level desired (0.1), the Bayesian method didn’t control the FDR at the desirable level, and generally produced higher FDR levels compared to the proposed method. We note that a multiplicity adjustment implemented for the frequentist p-values cannot be implemented for Bayesian posterior probabilities. The typical approach for adjusting the posterior probabilities uses the prior information and the Bayes Factor (see for instance [40]). Yet if such an adjustment would be implemented on the posterior probabilities, the FDR will naturally improve, but at the cost of reducing the power, which will then become lower than the power achieved by the proposed method (recall that with no calibration the power levels of the two methods are similar, as described in the Results Section). Thus, the conclusion may also be phrased as: “For equally powerful outcomes, the proposed method generated a substantially lower rate of false discoveries”.

**Table 2:** Power comparison between QTLBIM and the hierarchical testing approach.

Meta/Single genes	Num. of eQTL main effects	Epistasis effect	Power	
			QTLBIM	Hierarchical
single	2	−0.19	0.019	0.012
single	2	−0.20	0.023	0.023
single	2	−0.21	0.024	0.027
single	1	−0.19	0.017	0.016
single	1	−0.20	0.018	0.017
single	1	−0.21	0.020	0.025
single	0	−0.19	0.015	0.015
single	0	−0.20	0.018	0.017
single	0	−0.21	0.023	0.023
meta	2	−0.19	0.970	0.973
meta	2	−0.20	0.980	0.993
meta	2	−0.21	0.984	0.995
meta	1	−0.19	0.966	0.973
meta	1	−0.20	0.975	0.984
meta	1	−0.21	0.984	0.985
meta	0	−0.19	0.371	0.365
meta	0	−0.20	0.436	0.420
meta	0	−0.21	0.483	0.460

Simulation results. Standard error is <0.02 for power, <0.0007 for single traits and <0.0009 for meta-traits.

### Analysis of *Arabidopsis* data

The proposed two-stage search for epistasis was used for the analysis of the *Arabidopsis* data. First, the WGCNA hierarchical clustering was employed on the gene expression data for the purpose of building up multi-trait complexes (meta-traits). The TOM-based clustering followed by the Dynamic Tree Cut procedure discovered 314 trait clusters ranging from two to 1959 in size with mean of 23. Following the cluster identification, a meta-trait was generated for each cluster as the first principal component of the corresponding set of gene expression traits. The individual proportion of cluster variance explained by the corresponding meta-traits ranged between 0.2 and 0.93 with a median of 0.73.

Next, the two-step hierarchical testing procedure was employed. In the first step, a rough two-dimensional scan for epistasis was performed for each of the 314 meta-traits obtained in the previous step, using 47 sparse framework markers. Thus in the first step 1,081 pairs of markers were considered for each meta-trait, resulting in a total of 1,081*314 = 339,434 tests. Since the interest is in epistasis at all loci, epistatic effects attributed to either framework or secondary markers are of importance. Thus the rejection threshold should be chosen such that the full-tree FDR will be controlled. The multiplicative factor δ* was estimated by employing the hierarchical procedure repeatedly 10,000 times at level q = 0.1, resulting in an estimate of 1.015 (SE = 0.008). From (3), in order to control the full-tree FDR at level 0.1, q* = 0.1/2δ = 0.0472 was used. Twelve epistatic effects were identified at the first step. As can be seen in Table 2, the estimated contribution of epistasis to heritability varied between 1.6% and 10.3%. The two additive main effects were both significant in all cases except for case number 4, for which only one effect was significant.

**Figure 5:**
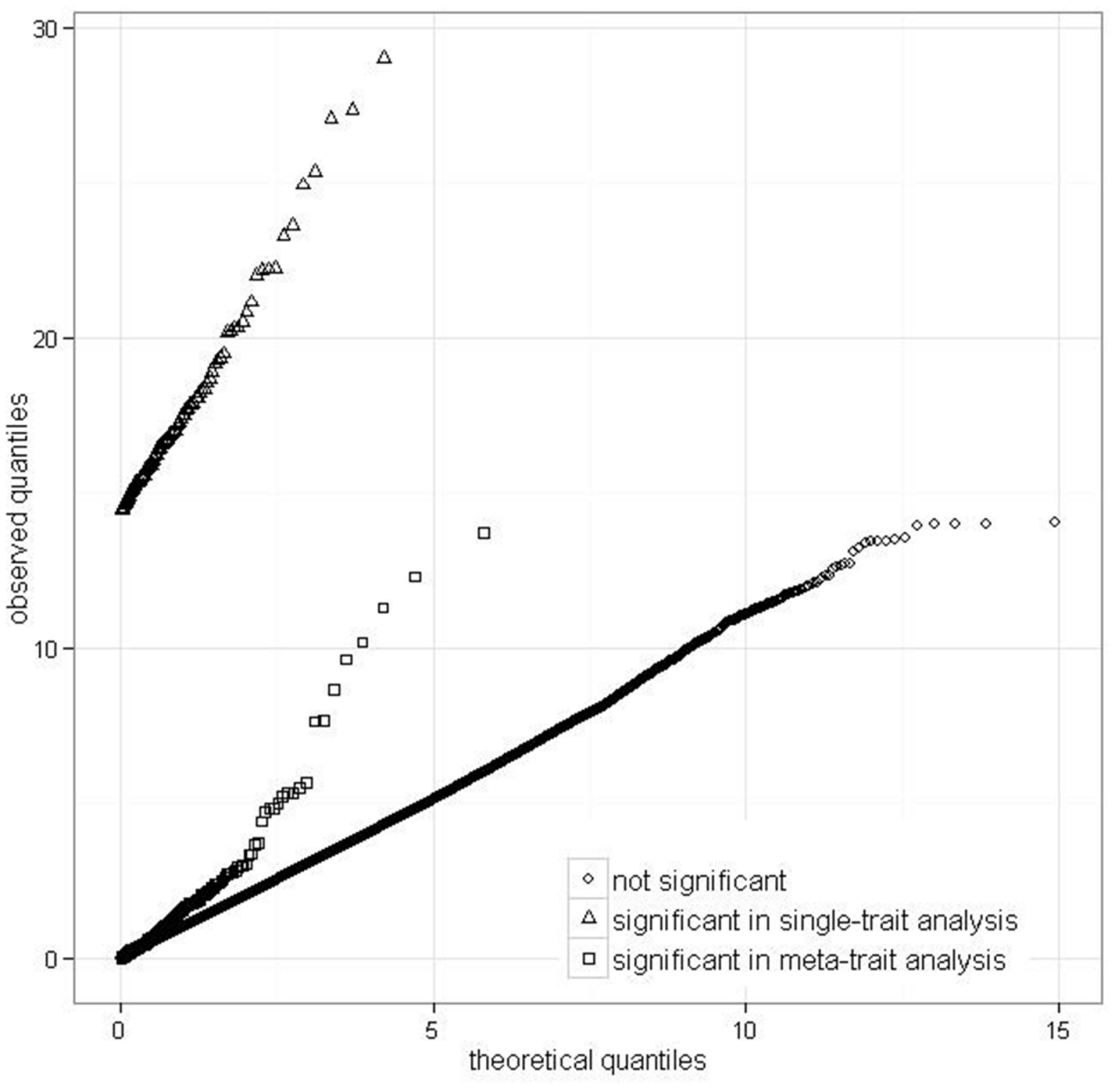
Q–Q-plot of –log(p-values) in single-trait analysis, assuming exponential distribution under the null. The p-values are for significance of epistatic interaction in the NOIA model. As expected, the p-values corresponding the null cases (marked by diamonds) follow a straight line that represents their distribution under the null. The p-values corresponding the presumably weak epistatic effects on meta-traits (marked by squares) generate p-values that are distinctly lower than the null p-values. The p-values corresponding the presumably strong epistatic effects on single-traits (marked by triangles) generate the lowest p-values.

In the second step, only the combinations of meta-trait and marker pair showing significant epistasis in the first step were tested for epistasis with increased resolution, using secondary markers neighboring the corresponding framework markers. The number of secondary marker pairs in the family of each identified framework marker-pair varied between 100 and 200, and the total number of tests in the second step was 1,673. For six families in the second step all hypotheses were rejected, and for the other five families, the proportion of discoveries varied between 0.6 and 0.97. In total, 1,506 epistatic effects were found in the second stage, summing up to 1,518 epistatic effects in total for both steps. The FDR control level can be obtained from (2), using 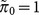:
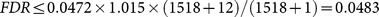



We examined what would be the results for the discovered effects on meta-traits, if instead the analysis was performed on single traits. Interestingly, the same pairwise effects on the corresponding single traits were not found significant. This may be explained by the poor testing power found by the simulation study for single traits. Yet, their p-values are clearly differentiated from those of all other non-significant effects on single traits (Fig. 5), as their distribution involves smaller values than the null distribution U(0,1). The significant effects involved 101 effects on expression of 75 single traits. Most of the effects included pairs of chromosomal intervals that were also involved in effects on meta-traits. The affected single traits did not show any correlation structure, which may explain why they were not discovered within meta-traits.

The markers associated with the detected epistatic effects are all concentrated in chromosomes 2, 4 and 5. Most epistatic effects are in sub-telomeric regions (telomers are repetitive DNA regions at the ends of chromosomes). Seven of the epistatic effects were found between eQTLs of two different chromosomes, and the remaining four were between two eQTLs of the same chromosome. As shown in Table 3, there are several genomic regions that are involved in more than one interaction. In addition, meta-traits 34 and 75, which represent 23 and 19 traits, respectively, were found to be affected by more than one pair of eQTL. It is noteworthy that only one of the twelve epistatic pairs included markers that have not been both simultaneously involved as main effect eQTLs as well (markers 267 and 490 identified in the initial scan, with only marker 267 identified also as a main effect eQTL). A schematic presentation of the results on a genomic map is provided within S1 Fig.

**Table 3:** Arabidopsis genome - detected epistasis effects.

Id	Meta-trait	Marker 1	Marker2	Heritability	
				Total	Due to epistasis
1	106	257	297	0.565	0.083
2	34	462	481	0.776	0.064
3	34	472	481	0.948	0.017
4	57	442	452	0.857	0.062
5	172	267	490	0.357	0.103
6	166	237	452	0.427	0.070
7	75	237	452	0.651	0.047
8	75	237	462	0.655	0.045
9	41	351	490	0.459	0.081
10	48	381	481	0.541	0.067
11	163	237	297	0.884	0.017
12	173	490	381	0.681	0.048

## Discussion

Epistasis is a genetic phenomenon that is challenging for detection within a genome-wide scan. It has been given relatively small attention until recently, even though it possesses a significant biological meaning and may contribute substantially to the understanding of complex gene networks that can lead to deciphering important biological mechanisms. This paper proposes a searching approach for epistasis effect on gene expression traits. It offers an analytical framework that, unlike other alternatives, combines the treatment of several important issues for the benefit of power gain and deeper biological interpretability. By taking advantage of the correlations between traits, the proposed two-stage algorithm is capable of identifying small epistatic effects which are otherwise challenging for detection, without relying on additional biological information. It performs an initial screen that is driven by the local dependence between markers, for potential epistatic regions. It guarantees control of the FDR at the desired level by using a procedure that accounts for the hierarchical testing structure generated by the screen. It shows advantage in power over similar procedures that do not include one of the key components of trait complexes and hierarchical testing. It allows flexibility in the choice of epistasis modeling approach, which should be in accordance with the interpretation of epistasis that is of interest for the researcher. The NOIA system, which is used in this study, models simple-to-interpret epistatic effects and can be tuned to match the type of population in hand.

A major strategy of the proposed approach is to reduce the very large number of tests for epistasis typical for a genome-wide scan. First, correlations between traits affected by epistasis are accounted for by considering unified representations of the expression quantitative traits. Aside from the reduction in the number of traits, a multi-trait complex (meta-trait) that is defined based on correlation may also provide a firmer base of evidence for phenotype-genotype relationships by relying on many traits altogether [33]. Furthermore, the identified effects also provide a broader picture on related co-regulation and network of genes [34]. Analysis of multi-trait complexes seems particularly beneficial for the case of small effects that are challenging to identify, since the chances to detect them is evidently higher compared to single trait analysis. It proved to be effective also for the alternative method we tested, the Bayesian model selection, which was not able to detect single genes effect, but gained substantial power when implemented on the multi-trait complexes. This finding is in consistence with the finding of [7] related to individual QTL effects. Second, similarities between the effects of neighboring loci are also accounted for by an initial screen for epistatic regions. The testing procedure controls the FDR while accounting for the hierarchical nature of the searching process.

The use of multi-trait complexes and the hierarchical search together achieves a dramatic reduction in the number of statistical tests. In the exemplifying analysis of the publicly available genome expression data for a RIL mapping population of the central plant model species *Arabidopsis thaliana*, a considerable reduction in the number of tests for epistasis was achieved by constructing 314 meta-traits by clustering expression data of 7,244 genes. Using the two-step hierarchical testing on the generated multi-trait complexes, 1,081*314+1,673 = 341,107 hypotheses were tested. If, instead, all possible combinations of markers and single traits were tested directly, namely 121,278 marker pairs for each one of the 7,244 traits, 878,537,832 tests would have been performed. Thus the overall number of tests was reduced by 2,575 folds.

A simulation study revealed the gain in both epistasis detection power and the contribution to heritability by the epistatic effects resulting from using multi-trait complexes rather than single traits. These observations are consistent with the initial findings of [7], [33], [35]. The case for which the performance measures obtained the lowest values was of epistasis with no main effects. These results seem to corroborate the findings in the early “deterministic sampling” study [33] regarding the detectability of two linked epistatic QTLs. The decrease in power at the presence of only epistasis may be explained by the relatively high residual variance characterizing a model containing only a weak epistatic effect. The PCA transformation seems to enhance the effect to a level that depends on the presence of other (main) effects, and the optimal configuration according to our findings was of one main effect. Thus it seems that certain relations between the number of main effect and the residual variance set the optimal conditions for identification of epistasis, Additional research is required for better understanding the impact of the model content on epistasis detection and the effectiveness of using the first principal component as a meta-trait may be further explored.

Cluster size may play an important role in epistasis identification. The obtained *Arabidopsis* meta-traits, of which variances were partly explained by epistasis, were based on clusters of a relatively small number of traits (5–23). This result may indicate that traits included in relatively large clusters are more likely to be related to each other by reasons other than being subjected together to the same epistatic effect, such as linkage and co-regulation of expression. Yet, they may also be affected by several different epistatic effects, in which case they must be further divided into sub-clusters, or alternatively the next principal components may be used.

The methodology used for the purpose of constructing multi-trait complexes based on the expression trait data should be a relatively minor stage. As many clustering techniques can be used, the chosen one must achieve high stability for the given data. The hierarchical clustering utilized in this study was based on a TOM-based dissimilarity measure and attained stability by merging a Dynamic Tree Cut algorithm [13]. This method has flexible parameterization and can be calibrated easily.

The hierarchical testing enabled control of the FDR across all tests for epistasis, thereby making our approach competitive in restraining the expected rate of false positive identifications. The hierarchical testing procedure achieved a considerable gain in power compared to direct testing of all combinations of marker-pairs and meta-traits. Under the dependence structure characterizing the tree of hypotheses used in this study, the FDR was controlled at a level lower than the boundary level established in [17] for independent hypotheses. The gain in both power and FDR control under some dependence is consistent with the findings of [19].

The implementation of the hierarchical testing scheme of the proposed approach may be further investigated in two aspects. First, the methods by which markers are chosen may take the genome-wide marker distribution into consideration. In this work, framework markers were chosen such that their regions will contain the same number of secondary markers. However, this may result in highly variable regional density of the secondary markers. An alternative strategy is also to account for distances between the markers. For example, it has been proposed to use biological information, such as locations with common mutation incidence, in order to choose the genotypic marker pairs for testing epistatic effects [4].

Second, the discovery of epistasis may depend also on the definition of epistasis within the statistical model. Indeed, some reports suggest that the way in which alleles interact is highly variable. For instance, the findings in this work suggest that several regions are involved in interactions with more than one region, and that the epistatic effects of one particular locus with several other loci may vary in form. In addition, for pairs of epistatic loci originating from two given regions, the corresponding effects sometimes may be of different form. Such effects were studied by [36], who inserted transposable elements into random positions within the bacterial genome, and found that fitness declined with increasing number of mutations. The fact that there was no evidence for effects of gene interaction was shown to result from the tendency of gene interactions to be highly variable and thus to even cancel each other out. Similarly, a study on epistasis in *Aspergillus niger* showed that fitness declined linearly with the number of mutations, without any evidence of epistasis on average [37]. However, genetic interactions explained variation in the reproductive success between different strains (see review in [3]). Both examples support the possibility that scanning for average epistatic effect may miss some of the effects that tend to cancel each other out. A possible alternative direction is to generalize the NOIA model, which tests for epistatic effects on an additive scale under the common linear regression assumptions. A more flexible modeling approach may achieve an improved fit, for instance by allowing (i) to define epistasis under a variable scale using a link function to exploratory variables, (ii) to consider non-monotonic effects (see [38] and [39]) and (iii) to account for differences in the marginal distributions of phenotypes.

Twelve combinations of meta-traits and framework marker pairs, all residing in *A. thaliana* chromosomes 2, 4 or 5, were identified as epistatic effect at the first step of the hierarchical search. For each of these marker pairs, most of the neighboring secondary markers were found to have an effect on the corresponding meta-trait. This further validates the already acknowledged regional association in functional roles of genomic sequences and supports an initial screen based on framework markers for the purpose of identifying epistatic eQTLs. Notably, one of the twelve framework marker pairs included a locus that has no main effect, thus strategies that test for epistasis only among pairs with main effects would fail to detect many of the effects identified here.

Single-trait eQTL analysis of the *Arabidopsis* data did not identify the effects corresponding to the ones identified in the analysis on meta-traits, but included marker pairs from the same DNA regions, which may be suspected as epistatic hotspots. The traits detected in single-trait analysis which also had strong epistatic effects were poorly correlated, while those that were not detected may have suffered a very low chance to be identified in a single trait analysis, as indicated by the simulation study for correlated single traits. These findings may suggest the existence of epistatic gene networks, having common epistatic effect and relatively small individual effects, as well as common involved chromosome regions. These are difficult to identify by a genome-wide association study due to low power. But analyzing them based on multi-trait complexes can dramatically increase the probability of their identification. Yet, the expression of some genes is not correlated with other expression traits but controlled by “individual” epistatic marker pairs that have a relatively large epistatic effect. These pairs can be effectively identified by single-trait analysis.

## Supporting Information

S1 Fig **A schematic representation of the epistatic findings on a genomic map – the Arabidopsis data.** Epistatic effects are shown on a partial map that includes only chromosomes found to contain epistasis (2, 4 and 5). An ellipse represents a meta-trait (of which id is specified on top), and the single traits it is based on are schematically shown inside. The ellipse is connected by two lines to two interacting markers located on the map. The vertical lines on the chromosomes mark the groups of “secondary” markers that make up the “framework” markers”. Each color corresponds to a particular epistatic effect on a particular meta-trait. The meta trait 75(19) was found to be affected by two pairs of markers that have one common marker (id 237). See Table 3 for the list of the twelve epistatic effects found and their heritability due to epistasis.
(TIF)
