# Supplementary figures and images for: Two-Stage Genome-Wide Search for Epistasis with Implementation to Recombinant Inbred Lines (RIL) Populations
Source: PLoS One. 2014 Dec 23;9(12):e115680. doi: 10.1371/journal.pone.0115680 (PMC4275240; doi:10.1371/journal.pone.0115680)

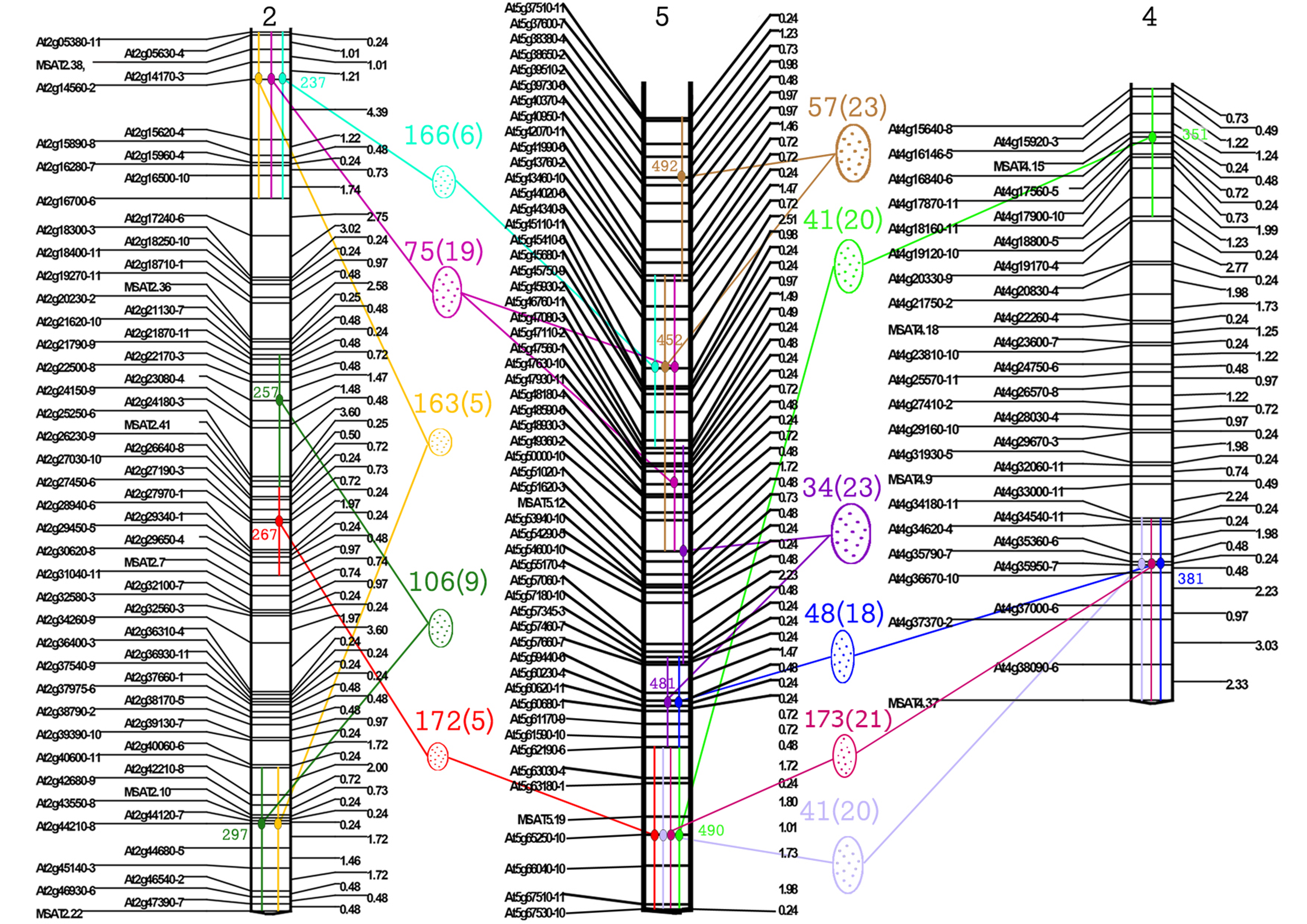

Supplement: S1 Fig — A schematic representation of the epistatic findings on a genomic map – the Arabidopsis data. Epistatic effects are shown on a partial map that includes only chromosomes found to contain epistasis (2, 4 and 5). An ellipse represents a meta-trait (of which id is specified on top), and the single traits it is based on are schematically shown inside. The ellipse is connected by two lines to two interacting markers located on the map. The vertical lines on the chromosomes mark the groups of “secondary” markers that make up the “framework” markers”. Each color corresponds to a particular epistatic effect on a particular meta-trait. The meta trait 75(19) was found to be affected by two pairs of markers that have one common marker (id 237). See Table 3 for the list of the twelve epistatic effects found and their heritability due to epistasis. (TIF) [file pone.0115680.s001.tif]
